# A Multivariate Polynomial Regression to Reconstruct Ground Contact and Flight Times Based on a Sine Wave Model for Vertical Ground Reaction Force and Measured Effective Timings

**DOI:** 10.3389/fbioe.2021.687951

**Published:** 2021-11-04

**Authors:** Aurélien Patoz, Thibault Lussiana, Bastiaan Breine, Cyrille Gindre, Davide Malatesta

**Affiliations:** ^1^ Institute of Sport Sciences University of Lausanne, Lausanne, Switzerland; ^2^ Research and Development Department Volodalen Swiss Sport Lab, Aigle, Switzerland; ^3^ Research and Development Department Volodalen, Chavéria, France; ^4^ Research Unit EA3920 Prognostic Markers and Regulatory Factors of Cardiovascular Diseases and Exercise Performance Health Innovation Platform University of Franche-Comté, Besançon, France; ^5^ Department of Movement and Sports Sciences Ghent University, Ghent, Belgium

**Keywords:** running, biomechanics, sensors, inertial measurement unit, machine learning

## Abstract

Effective contact ( $t_{ce}$ ) and flight ( $t_{fe}$ ) times, instead of ground contact ( $t_{c}$ ) and flight ( $t_{f}$ ) times, are usually collected outside the laboratory using inertial sensors. Unfortunately, $t_{ce}$ and $t_{fe}$ cannot be related to $t_{c}$ and $t_{f}$ because the exact shape of vertical ground reaction force is unknown. However, using a sine wave approximation for vertical force, $t_{ce}$ and $t_{c}$ as well as $t_{fe}$ and $t_{f}$ could be related. Indeed, under this approximation, a transcendental equation was obtained and solved numerically over a $t_{ce}\text{ x}\;t_{fe}$ grid. Then, a multivariate polynomial regression was applied to the numerical outcome. In order to reach a root-mean-square error of 0.5 ms, the final model was given by an eighth-order polynomial. As a direct application, this model was applied to experimentally measured $t_{ce}$ values. Then, reconstructed $t_{c}$ (using the model) was compared to corresponding experimental ground truth. A systematic bias of 35 ms was depicted, demonstrating that ground truth $t_{c}$ values were larger than reconstructed ones. Nonetheless, error in the reconstruction of $t_{c}$ from $t_{ce}$ was coming from the sine wave approximation, while the polynomial regression did not introduce further error. The presented model could be added to algorithms within sports watches to provide robust estimations of $t_{c}$ and $t_{f}$ in real time, which would allow coaches and practitioners to better evaluate running performance and to prevent running-related injuries.

## Introduction

Ground contact $\left(t_{c}\right)$ and flight $\left(t_{f}\right)$ times are key temporal parameters of running biomechanics. Indeed, Novacheck (1998) postulated that the presence of $t_{f}$ allowed distinguishing walking from running gaits. In other words, the duty factor (the ratio of $t_{c}$ over stride duration) is under 50% for running (Minetti, 1998; Folland et al., 2017). Moreover, $t_{c}$ was shown to be self-optimized to minimize the metabolic cost of running (Moore et al., 2019). These two parameters are obtained from foot-strike (FS) and toe-off (TO) events. More specifically, $t_{c}$ represents the time from FS to TO of the same foot, while $t_{f}$ is the time from TO of one foot to FS of the contralateral foot. Therefore, $t_{c}$ and $t_{f}$ rely on the accuracy of FS and TO detections, for which the use of force plates is considered the gold standard method. However, force plates could not always be available and used (Abendroth-Smith, 1996; Maiwald et al., 2009). In such case, alternatives would be to use a motion capture system (Lussiana et al., 2019; Patoz et al., 2020) or a light-based optical technology (Debaere et al., 2013). Nevertheless, even though these three systems can be used outside the laboratory (Purcell et al., 2006; Hébert-Losier et al., 2015; Ammann et al., 2016; Lussiana and Gindre, 2016), they suffer a lack of portability and are restricted to a specific and small capture volume, that is, they do not allow continuous temporal gait data collection throughout the entire training or race. To overcome such limitations, techniques to identify FS and TO events were developed using portative tools such as inertial measurement units (IMUs), which are easy to use, low cost, and suitable for field measurements and very practical to use in a coaching environment (Camomilla et al., 2018).

Different techniques to identify gait events are available and depend on the placement of the IMU on the human body (Moe-Nilssen, 1998; Lee et al., 2010; Flaction et al., 2013; Giandolini et al., 2014; Norris et al., 2014; Giandolini et al., 2016; Gindre et al., 2016; Falbriard et al., 2018; Falbriard et al., 2020). Among them, when the IMU is positioned near the sacrum, that is, close to the center of mass, the vertical acceleration signal can be used to determine effective contact $\left(t_{ce}\right)$ and flight $\left(t_{fe}\right)$ times, instead of $t_{c}$ and $t_{f}$ (Flaction et al., 2013; Gindre et al., 2016). To delineate these effective timings, the vertical force is calculated based on Newton’s second law using the body mass (*m*) of individuals and the vertical acceleration data. Then, these effective timings are based on effective FS (eFS) and effective TO (eTO) events. More precisely, eFS and eTO correspond to the instants of time where the vertical force increases above and decreases below body weight (*mg*), respectively (Cavagna et al., 1988). The authors (Flaction et al., 2013; Gindre et al., 2016) did not mention why a 20 N threshold was not used to determine FS and TO events from their IMU data, even though this is the reference when using force plates data for event detection (Smith et al., 2015). However, the vertical acceleration recorded by an IMU during $t_{f}$ is usually negative (Gindre et al., 2016), while a force plate measure gives exactly zero. Therefore, it could be suspected that a 20 N threshold would not be reliable to obtain FS and TO events when dealing with IMU data, while the time at which the vertical force is equal to body weight would be equivalent between IMU and force plate data.

Using effective timings or $t_{c}$ and $t_{f}$ provide the same step duration, that is, it is given by either the sum of $t_{c}$ and $t_{f}$ or $t_{ce}$ and $t_{fe}$ . Thus, this temporal information is not lost. As for the effect of running speed, $t_{ce}$ and $t_{c}$ both decrease with increasing running speed, even though the decrease is much more important for $t_{c}$ than $t_{ce}$ (Cavagna et al., 2008; Da Rosa et al., 2019). Concerning $t_{fe}$ and $t_{f}$ , their trend with increasing running speed is not similar. Indeed, $t_{fe}$ tends to slightly decrease, while $t_{f}$ increases almost up to a plateau with increasing running speed (Cavagna et al., 2008; Da Rosa et al., 2019). In addition, $t_{ce}$ and $t_{fe}$ cannot directly be related to $t_{c}$ and $t_{f}$ , the reason being that the fraction of time spends below body weight during $t_{c}$ depends on the shape of the vertical ground reaction force, which is not precisely known when using IMUs (see above). Thus, $t_{c}$ and $t_{f}$ , parameters that are directly related to them, for example, duty factor (Minetti, 1998; Folland et al., 2017), as well as parameters that can be estimated from them, for example, vertical oscillation and vertical stiffness (Morin et al., 2005), cannot be obtained. Hence, the assessment of running biomechanics is restricted when using $t_{ce}$ and $t_{fe}$ .

**FIGURE 1:**
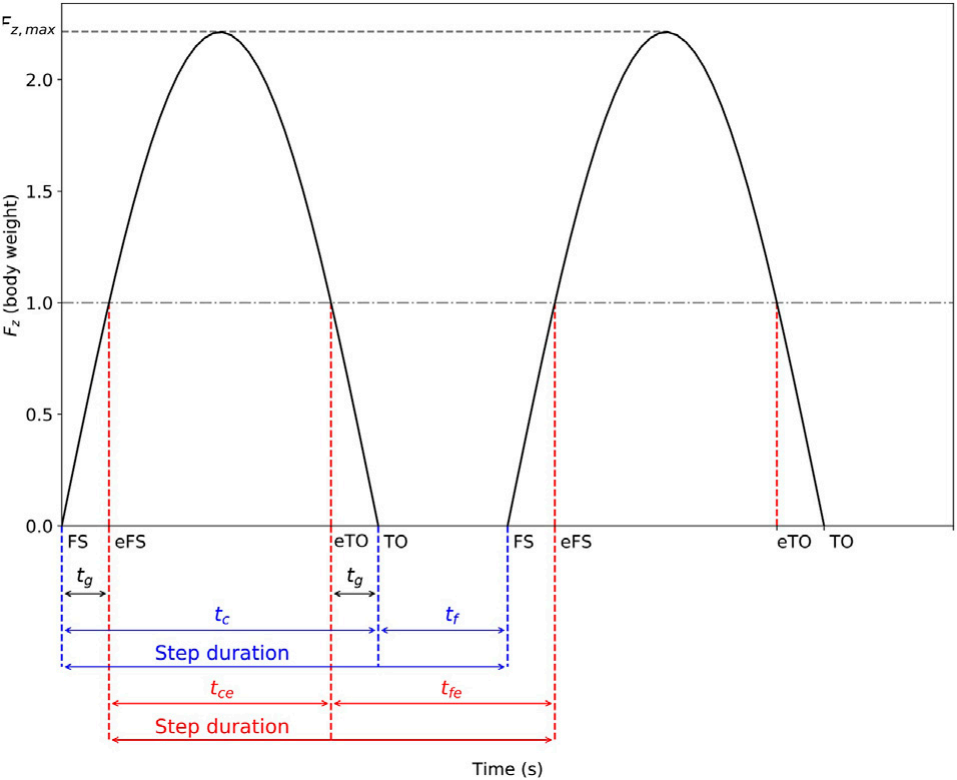
Vertical ground reaction force $\left(F_{z}\right)$ under the sine wave approximation, peak vertical force $\left(F_{z,\max}\right)$ , foot-strike (FS) and toe-off (TO) events together with their corresponding effective events (eFS and eTO), as well as contact $\left(t_{c}\right)$ , flight $\left(t_{f}\right)$ , effective contact $\left(t_{ce}\right)$ , and effective flight $\left(t_{fe}\right)$ times, and time to reach body weight $\left(t_{g}\right)$ , for a typical running stride. Noteworthy, step duration is the same when using effective or usual timings.

Nonetheless, the vertical ground reaction force can be approximated using a sine wave as $F_{z}\left(t\right)=F_{z,\max}\sin\left(\pi t/t_{c}\right)$ , where, based on momentum conservation law, $F_{z,\max}=mg\pi \left(t_{f}/t_{c}+1\right)/2$ (Alexander, 1989; Kram and Dawson, 1998; Dalleau et al., 2004; Morin et al., 2005). In such case, the vertical ground reaction force is symmetric around $t_{c}/2$ , which means that the time duration between FS and eFS as well as between eTO and TO, called $t_{g}$ in what follows, are the same. Thereby, under the sine wave assumption, $t_{c}$ and $t_{f}$ can be obtained from $t_{ce}$ and $t_{fe}$ using $t_{c}=t_{ce}+2t_{g}$ and $t_{f}=t_{fe}-2t_{g}$ , if $t_{g}$ is known. These timings and the sine wave vertical ground reaction force are depicted in Figure 1 for a typical running stride. Recognizing that $F_{z}\left(t_{g}\right)=mg=F_{z,\max}\sin\left(\frac{\pi t_{g}}{t_{c}}\right)$ , and using the definition of $F_{z,\max}$ given before, the following equation is obtained:

$$\csc\left(\frac{\pi t_{g}}{t_{ce}+2t_{g}}\right)=\frac{\pi }{2}\left(\frac{t_{fe}-2t_{g}}{t_{ce}+2t_{g}}+1\right),$$ (1)

which could not be solved analytically for $t_{g}$ (transcendental equation; Supplementary File) using Mathematica v12.1 (Wolfram, Oxford, UK), that is, no closed-form solution exists. Therefore, a numerical solution is required for any pair of $t_{ce}$ and $t_{fe}$ . Ultimately, a mathematical modeling of $t_{g}$ over the numerical $t_{ce}$ x $t_{fe}$ grid could be performed, and its accuracy could be evaluated using advanced data analysis techniques like machine learning. Indeed, supervised machine learning models like linear regressions have been used to model relationships between biomechanical measures and clinical outcomes (Halilaj et al., 2018; Backes et al., 2020; Alcantara et al., 2021). However, to the best of our knowledge, no attempt to provide such a model equation for $t_{g}$ has been made so far.

Hence, the purpose of this study was to obtain a mathematical modeling of $t_{g}$ under the sine wave approximation of the vertical ground reaction force so that $t_{c}$ and $t_{f}$ can be reconstructed from $t_{ce}$ and $t_{fe}$ . As a direct experimental application, the proposed model was applied to experimentally measured $t_{ce}$ values. Then, the reconstructed $t_{c}$ values were compared to their corresponding experimental ground truth (gold standard).

## Materials and Methods

### Numerical Analysis

Brent’s method (also known as van Wijngaarden Dekker Brent method) (Brent, 1973; Press et al., 1992) was used to find the zeros of Eq. 1 for any pair of $t_{ce}$ and $t_{fe}$ . The zero of interest for a given $t_{ce}$ and $t_{fe}$ pair was considered to lie between 0 and the minimum of Eq. 1, which was minimized using the Broyden Fletcher Goldfarb Shanno method (Broyden, 1970; Fletcher, 1970; Goldfarb, 1970; Shanno, 1970). The numerical analysis was carried out using $t_{ce}$ and $t_{fe}$ values varying between 2.5 and 505 ms and using a grid spacing of 7.5 ms (4,624 grid points). The grid limits were chosen due to the fact that running requires 1) both a ground contact and a flight phase, that is, $t_{ce}$ and $t_{fe}$ cannot be 0 and 2) $t_{c}$ belongs to the interval [100 ms, 400 ms] and $t_{f}$ belongs to the interval [0 ms,250 ms] (Cavagna et al., 2008; Da Rosa et al., 2019; Lussiana et al., 2019), and to include any atypical $t_{ce}$ and $t_{fe}$ pair, that is, atypical runners. Noteworthy, the justification of the grid spacing is provided in Appendix. The grid spacing was dependent on the error threshold set to the mathematical modeling.

### Mathematical Modeling

#### Boundary Relationship Between $t_{ce}$ and $t_{fe}$

**FIGURE 2:**
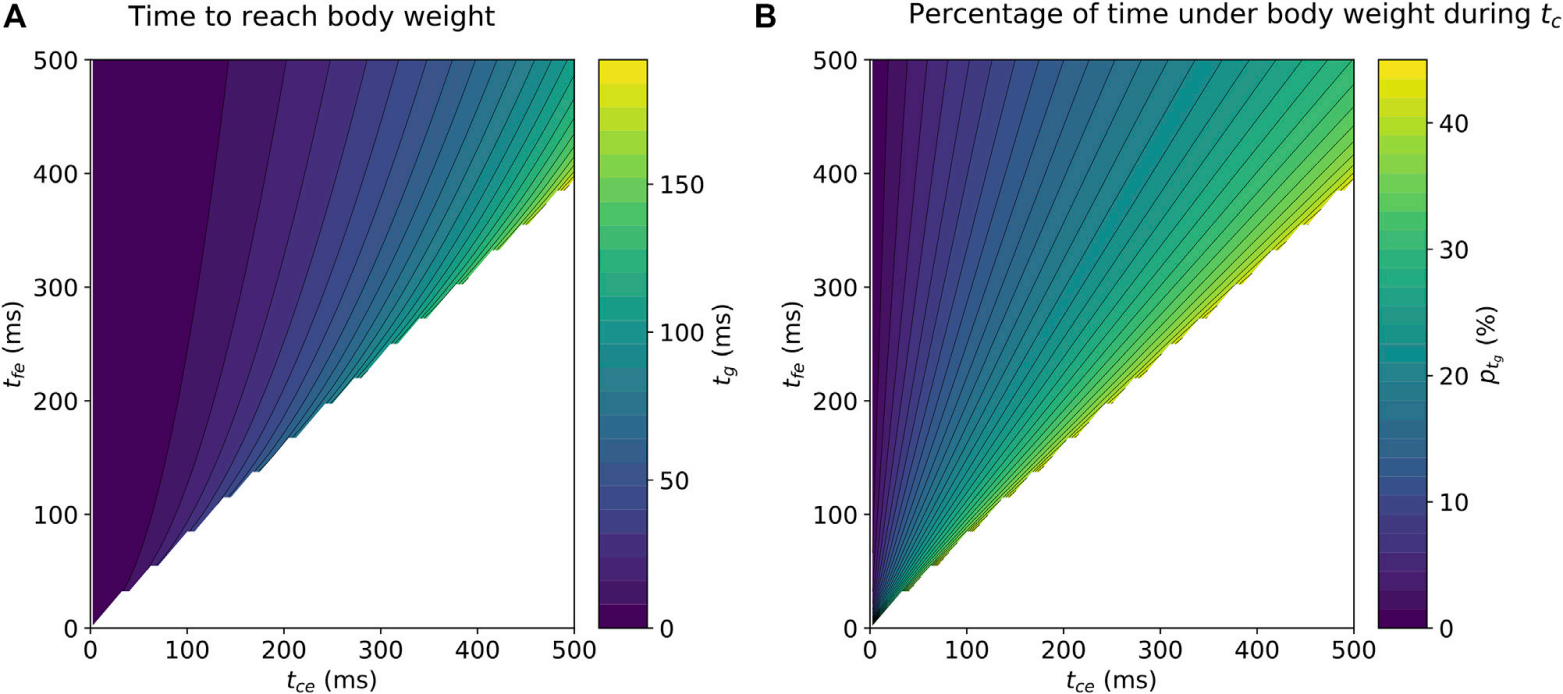
Contour plots depicting a) the numerically calculated time $\left(t_{g}\right)$ necessary to reach body weight and B) the corresponding percentage (*p*_*t_g_*_) of time under body weight during ground contact time $\left(t_{c}\right)$ . The numerical simulation assumed a sine wave model for vertical ground reaction force and was performed over the $t_{ce}$ x $t_{fe}$ grid.

The numerical analysis showed that a linear boundary relationship is present between $t_{ce}$ and $t_{fe}$ (see Results Figure 2), that is, there is no solution for $t_{g}$ if $t_{fe}$ is higher than a certain percentage of $t_{ce}$ . This boundary relationship was computed by extracting the boundary points, that is, the smallest existing $t_{fe}$ values for every $t_{ce}$ grid point (68 pair of points). Then, a linear regression using ordinary least square was performed on a training set consisting of 85% of the entire set of boundary points. The *y-*intercept of the fitted linear model was held fixed at 0, the reason being that a null $t_{ce}$ necessarily ensures a null $t_{fe}.$ The linear model was tested on the remaining 15% points (testing set) and evaluated using the coefficient of determination $\left(R^{2}\right)$ and root-mean-square error (RMSE).

#### Modeling a $t_{g}$ Surface as Function of $t_{ce}$ and $t_{fe}$

The numerical analysis showed that $t_{g}$ could be described by a smoothly increasing surface when increasing $t_{ce}$ and $t_{fe}$ (see Results Figure 2). Therefore, a multivariate polynomial regression using ordinary least square was performed on a training set consisting of $t_{g}$ values corresponding to 85% of the points within the boundary limits (i.e., the non-discarded grid points). The regression was performed using polynomials of order 1 to 15 and including intercept and interaction terms. RMSE on the remaining 15% points (testing set) was computed for each fitted polynomial.

### Experimental Application

#### Participant Characteristics

One hundred recreational runners (Honert et al., 2020), 75 males (age: 31 ± 8 years, height: 180 ± 6 cm, body mass: 70 ± 7 kg, and weekly running distance: 37 ± 24 km) and 25 females (age: 30 ± 7 years, height: 169 ± 5 cm, body mass: 61 ± 6 kg, and weekly running distance: 20 ± 14 km), voluntarily participated in the present study. For study inclusion, participants were required to be in good self-reported general health with no current or recent lower extremity injury (≤1 month), to run at least once a week, and to have an estimated maximal aerobic speed ≥14 km/h. The study protocol was approved by the Ethics Committee (CER-VD 2020–00334) and adhered to the latest Declaration of Helsinki of the World Medical Association.

#### Experimental Procedure

After providing written informed consent, each participant performed a 7-min warm-up run on an instrumented treadmill (Arsalis T150—FMT-MED, Louvain-la-Neuve, Belgium). Speed was set to 9 km/h for the first 3 min and was then increased by 0.5 km/h every 30 s. This was followed, after a short break (<5 min), by three 1-min runs (9, 11, and 13 km/h) performed in a randomized order (1-min recovery between each run). 3D kinetic data were collected during the first 10 strides following the 30-s mark of running trials. All participants were familiar with running on a treadmill as part of their usual training program and wore their habitual running shoes.

### Data Collection

3D kinetic data (1,000 Hz) were collected using the force plate embedded into the treadmill and using Vicon Nexus software v2.9.3 (Vicon, Oxford, UK). The laboratory coordinate system was oriented such that *x*-, *y*-, and *z*-axes denoted mediallateral (pointing toward the right side of the body), posterioranterior, and inferiorsuperior axis, respectively. Ground reaction force (analog signal) was exported in .c3d format and processed in Visual3D Professional software v6.01.12 (C-Motion Inc, Germantown, MD, United States). 3D ground reaction force signal was low-pass–filtered at 20 Hz using a fourth-order Butterworth filter and down-sampled to 200 Hz to represent a sampling frequency corresponding to typical measurements recorded using a central inertial unit.

### Data Analysis

For each running trial, eFS and eTO events were identified within Visual3D by applying a body weight threshold to the *z-*component of the ground reaction force (Cavagna et al., 1988). More explicitly, eFS was detected at the first data point greater or equal to *mg* within a running step, while eTO was detected at the last data point greater or equal to *mg* within the same running step. $t_{ce}$ and $t_{fe}$ were defined as the time from eFS to eTO of the same foot and from eTO of one foot to eFS of the contralateral foot, respectively.

In addition, FS and TO events were also identified within Visual3D. These events were detected by applying a 20 N threshold to the *z-*component of the ground reaction force (Smith et al., 2015). More explicitly, FS was detected at the first data point greater or equal to 20 N within a running step, while TO was detected at the last data point greater or equal to 20 N within the same running step. $t_{c}$ and $t_{f}$ were defined as the time from FS to TO of the same foot and from TO of one foot to FS of the contralateral foot, respectively.

The recorded vertical ground reaction force permitted to precisely measure $t_{c}$ and $t_{f}$ as well as $t_{ce}$ and $t_{fe}$ . Then, each $t_{ce}$ and $t_{fe}$ pair was fed to the best multivariate polynomial model to compute $t_{g}$ , which ultimately allowed to obtain $t_{c}$ . An instrumented treadmill was used to measure $t_{ce}$ and $t_{fe}$ (gold standard), instead of an IMU to remove any potential measurement error that would come from the IMU itself. Hence, the error obtained when comparing the reconstructed $t_{c}$ (obtained using the mathematical model and $\;t_{ce}$ and $t_{fe}$ ) to its corresponding experimental ground truth (obtained from FS and TO events) could solely be coming from the sine wave assumption and the mathematical modeling but not from the measurement of $t_{ce}$ and $t_{fe}$ .

### Statistical Analysis

All data are presented as mean ± standard deviation. The reconstructed $t_{c}$ values were compared to corresponding experimental ground truth $t_{c}$ values using a BlandAltman plot (Bland and Altman, 1995; Atkinson and Nevill, 1998). Noteworthy, as step time is conserved, differences between measured and reconstructed $t_{f}$ values depicted the opposite behavior compared with the differences between measured and reconstructed $t_{c}$ values.

Systematic bias, lower and upper limit of agreements, and 95% confidence intervals (CI) were computed as well as RMSE. The difference between reconstructed and ground truth $t_{c}$ values was quantified using Cohen’s *d* effect size and interpreted as very small, small, moderate, and large when |*d*| values were close to 0.01, 0.2, 0.5, and 0.8, respectively (Cohen, 1988). Statistical analysis was performed using Jamovi (v1.2, retrieved from https://www.jamovi.org), with the level of significance set at *p* ≤ 0.05.

## Results

### Numerical Analysis

The numerically calculated $t_{g}$ values over the $t_{ce}$ x $t_{fe}$ grid are provided in Figure 2A, while Figure 2B depicts the corresponding percentage of time $\left(p_{t_{g}}\right)$ spent under body weight during $t_{c}$ , $\left[p_{t_{g}}=100*2t_{g}/(t_{ce}+2t_{g})\right]$ .

### Mathematical Modeling

#### Boundary Relationship Between ${\text{t}}_{\text{ce}}$ and ${\text{t}}_{\text{fe}}$

The linear regression gave the model (Eq. 2):

$$t_{fe}=0.795\;t_{ce}.$$ (2)

**FIGURE 3:**
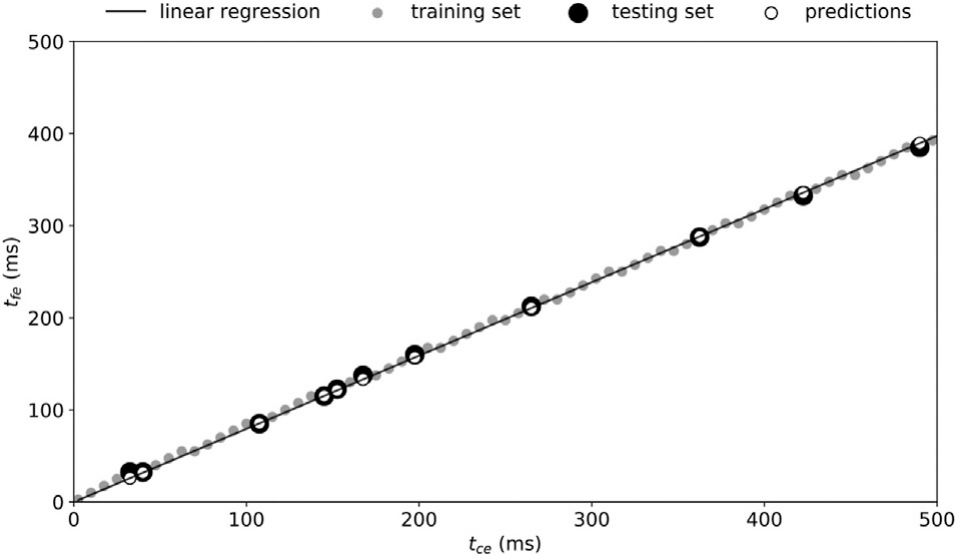
Boundary relationship between $t_{ce}$ and $t_{fe}$ . A linear regression (solid line) was obtained using 85% of the entire boundary points (training set, small gray dots) and validated on the remaining 15% points (testing set, large black dots). Predictions are given by the black circles and led to a root-mean-square error of 3.2 ms $\left(R^{2}=99.9\%\right)$ .

Applying this model to the testing set led to an $R^{2}$ of =99.9% and RMSE of 3.2 ms. The linear regression, training, and testing sets as well as predicted values are depicted in Figure 3.

#### Modeling a $t_{g}$ Surface as Function of $t_{ce}$ and $t_{fe}$

**FIGURE 4:**
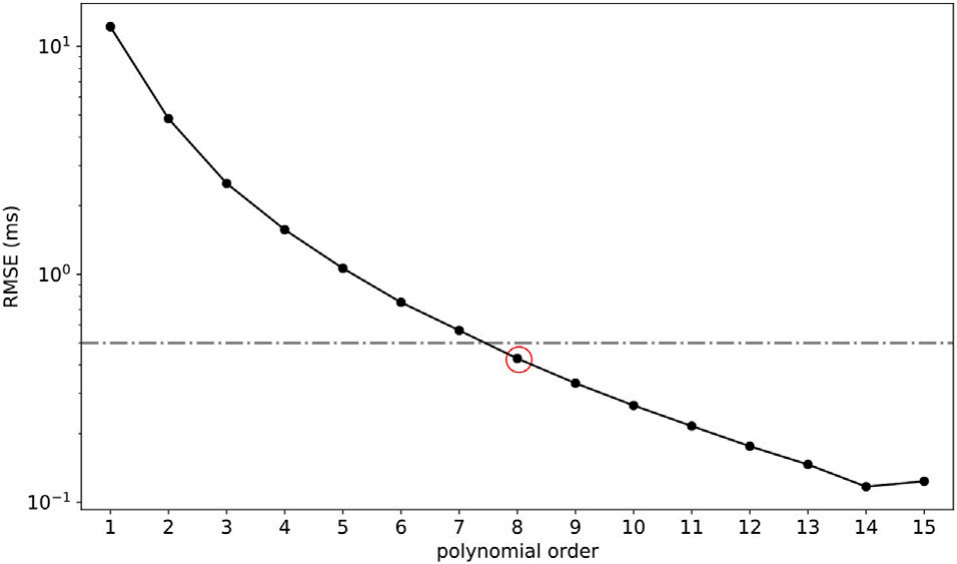
Root-mean-square error (RMSE) computed on the testing set (15% points) for polynomial fits of order 1 to 15 performed on the training set (85% points). The red circle denotes the final model of choice, an eighth-order polynomial model (RMSE = 0.43 ms; $R^{2}=99.99\%$ ), and the gray line depicts the RMSE threshold of 0.5 ms.

The grid points which did not satisfy the previously obtained boundary relationship (Eq. 2) were discarded (1814 discarded points). RMSE computed for each multivariate polynomial regression (order 1–15) is depicted in Figure 4. The polynomial which provided an RMSE smaller than 0.5 ms was kept as the final model of choice (RMSE = 0.43 ms; $R^{2}=99.99\%$ ) and corresponded to a polynomial model including up to eighth-order terms [ $P_{8}\left(t_{ce},t_{fe}\right)$ , Eq. 3]. The coefficients ( $\alpha_{ij}$ , where 0≤i+j≤8 ) of the multivariate polynomial model are given in Table 1.

$$P_{8}\left(t_{ce},t_{fe}\right)=\sum_{i=0}^{8}\sum_{j=0}^{8-i}\alpha_{i,j}t_{ce}^{i}t_{fe}^{j}$$ (3)

**TABLE 1:** Coefficients ( $\alpha_{ij}$ , where 0≤i+j≤8 ) of the eighth-order multivariate polynomial model given by Eq. 3.

		$j\left(\mathrm{exponent}\;\mathrm{of}\;t_{\mathrm{fe}}\right)$								
		0	1	2	3	4	5	6	7	8
$j\left(\mathrm{exponent}\;\mathrm{of}\;t_{fe}\right)$	0	−5.17E-5	−6.18E-2	2.73E0	−4.41E1	3.532	−1.55E3	3.783	−4.83E3	2.513
	1	2.84E-1	−1.41E1	2.64E2	−2.45E3	1.234	−3.38E4	4.834	−2.78E4	
	2	1.17E1	−3.12E2	3.91E3	−2.49E4	8.434	−1.43E5	9.534		
	3	8.26E1	−2.25E3	2.20E4	−1.01E5	2.155	−1.72E5			
	4	5.13E2	−9.73E3	6.68E4	−1.90E5	1.865				
	5	1.63E3	−2.32E4	9.82E4	−1.22E5					
	6	3.41E3	−2.76E4	4.65E4						
	7	3.15E3	−8.66E3							
	8	4.62E2								

**FIGURE 5:**
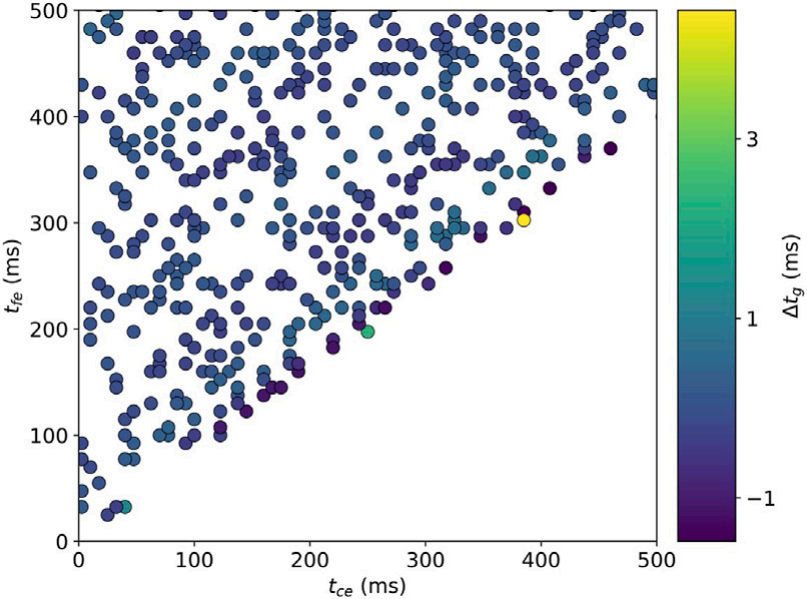
Differences between $t_{g}$ values $\left(\Delta t_{g}\right)$ computed numerically (Section 2) and using the eighth-order polynomial model for the testing set (15% points). A difference larger than 2 ms was depicted for only two points (green and yellow circles) in the testing set, which were close to the boundary limit.

Noteworthy, the threshold on RMSE ensured an error smaller than 1 ms on the reconstructed $t_{c}$ . The differences between $t_{g}$ values computed numerically and using the eighth-order polynomial model for the testing set (15% points) are depicted in Figure 5.

### Experimental Application

**FIGURE 6:**
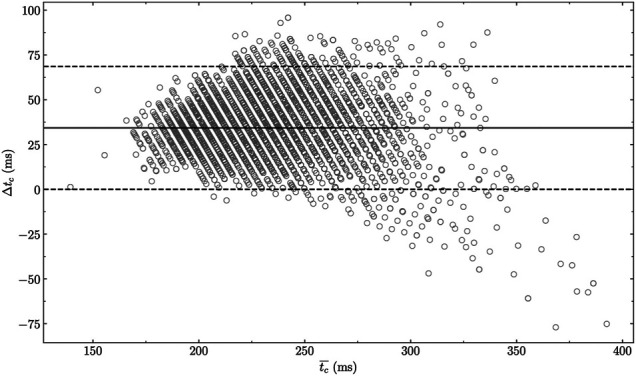
BlandAltman plot comparing experimentally measured and reconstructed $t_{c}$ using the multivariate polynomial model given by Eq. 3, which reports a systematic bias of 34.3 ms (95% confidence intervals [33.8 ms, 34.7 ms]). $\text{Δ}t_{c}$ : measured $t_{c}$ − reconstructed $t_{c}$ and $\bar{t_{c}}$ : average of measured and reconstructed $t_{c}$ .

Reconstructed $t_{c}$ values were compared to corresponding experimental ground truth $t_{c}$ values using a BlandAltman plot, which is depicted in Figure 6. A systematic positive bias of 34.3 ms (95% CI [33.8 ms, 34.7 ms]) was obtained. The lower and upper limits of agreements were 0.0 ms (95% CI [−0.8 ms, 0.8 ms]) and 68.6 ms (95% CI [67.8 ms, 69.3 ms]), respectively. The RMSE between reconstructed and measured $t_{c}$ was 38.5 ms (7.6%), and Cohen’s *d* effect size was large (*d* = 1.1).

## Discussion

The proposed eighth-order multivariate polynomial model (Eq. 3) could be used to obtain $t_{c}$ and $t_{f}$ when an IMU is used to measure $t_{ce}$ and $t_{fe}$ . Thereby, important parameters to assess running biomechanics such as duty factor (Lussiana et al., 2019; Patoz et al., 2020), as well as vertical oscillation and vertical stiffness (Morin et al., 2005), could be calculated more precisely. Having these parameters would allow coaches and practitioners to better evaluate running performance outside the laboratory such as in a coaching environment and during an entire training or race, and to prevent running-related injuries.

In the case where an algorithm based on effective timings is running on the fly to provide live feedbacks, such as in sports watches, one could simply add the proposed model in the end of the algorithm chain, right before computing the biomechanical outcomes. However, many operations should be performed in a very small amount of time, where the number of operations is directly related to the order of the polynomial. Indeed, knowing that the number of terms in an $n^{\text{th}}$ -order polynomial composed of two variables is given by $C_{2}^{n+2}$ , then $C_{2}^{n+2}-3$ calculations are required to compute the polynomial features, that is, $t_{ce}^{i}$ and $t_{fe}^{i}$ , where 2≤i≤n. In addition, $C_{2}^{n+2}-1$ multiplications and $C_{2}^{n+2}-1$ additions are necessary to calculate $t_{g}$ . Therefore, such a large number of operations could be problematic for the small computing power available in sports watches. If this is really an issue, the order of the polynomial could be decreased. For instance, a third-order polynomial model gave an RMSE of 2.5 ms (Figure 4), which, depending on the application, might already be sufficient. In this case, the number of operations would be reduced from 130 (eighth order) to 25 (third order), leading to a 5 times speedup, assuming sequential calculations (no parallelization).

**FIGURE 7:**
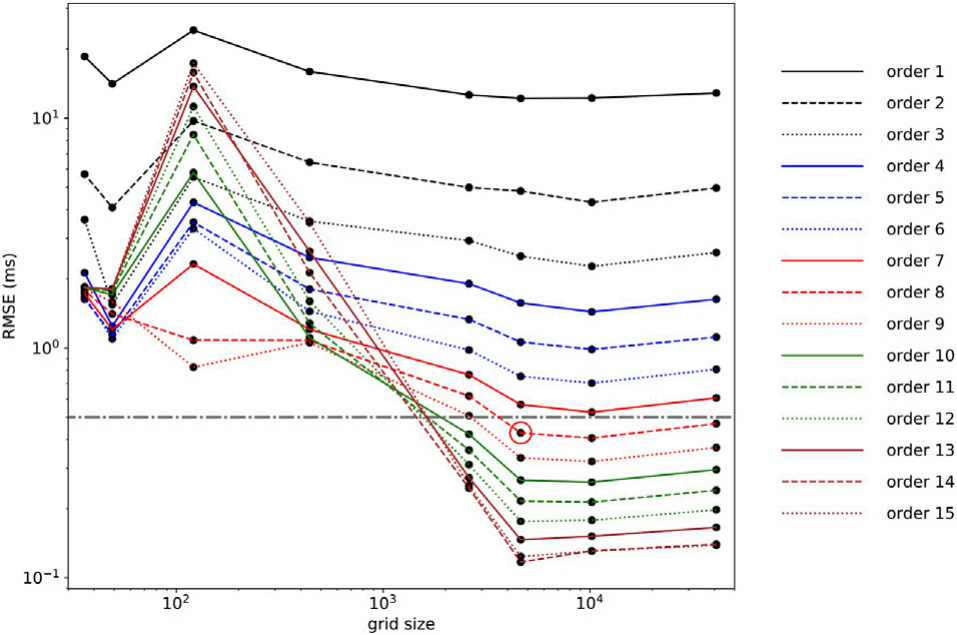
Root-mean-square error as a function of grid size ranging from 36 to 40,804 total points and for each polynomial regression (1st to 15th order). The red circle denotes RMSE corresponding to a polynomial (eighth order) chosen in Section 3.2 (0.43 ms), and the gray line depicts an RMSE threshold of 0.5 ms.

The multivariate polynomial model (Eq. 3) was applied to experimentally measured $t_{ce}$ values. These results permitted us to show that the experimental ground truth $t_{c}$ was, on average, 34.3 ms higher than the reconstructed one. Since the multivariate polynomial regression reported an RMSE of 0.43 ms, the large systematic bias obtained here was inherently due to the sine wave approximation of the vertical ground reaction force. To further justify the previous statement, the polynomial depicting the smallest RMSE, that is, the 14th-order polynomial (RMSE = 0.12 ms; Figure 7), was used to compute $t_{c}$ based on $t_{ce}$ . Doing so, the following results were obtained: RMSE = 38.6 ms (7.6%), *d* = 1.1 (large effect size), and systematic bias = 34.2 ms [95% CI (33.7 ms, 34.6 ms)]. Therefore, to go beyond the scope of this study, future research should focus on defining a more accurate model of the vertical ground reaction force. Indeed, the sine wave approximation constituted the main limitation of the novel multivariate polynomial model proposed in this study.

## Conclusion

To conclude, in the present study, an eighth-order multivariate polynomial model was constructed based on the numerical solution of the transcendental equation given by Eq. 1. The proposed model permitted to compute $t_{c}$ and $t_{f}$ from effective timings $\left(t_{ce}\;\text{and}\;t_{fe}\right)$ using the sine wave approximation of the vertical ground reaction force. The model was chosen so that RMSE was smaller than 0.5 ms. Therefore, the error in the computation of $t_{c}$ and $t_{f}$ was coming from the sine wave approximation, while the polynomial regression did not introduce further error.

## Figures and Tables

**FIGURE A1:**
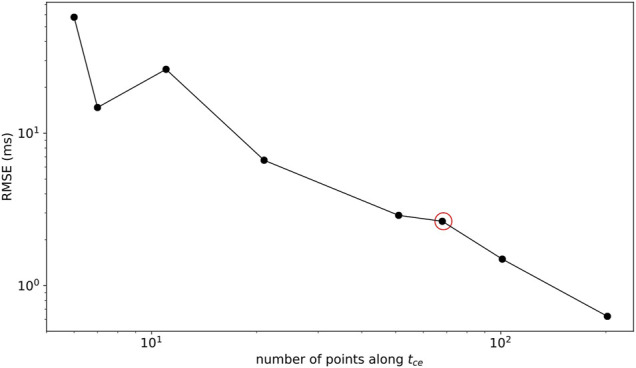
Root-mean-square error (RMSE) as a function of the number of points along tce and ranging from 6 to 202. The red circle denotes RMSE corresponding to the boundary relationship computed in Section 3.1 (3.2 ms).

## Data Availability

The raw data supporting the conclusion of this article will be made available by the authors, without undue reservation.

## Appendix: Justification of the Choice of the $t_{ce}$ x $t_{fe}\;$ Grid

To justify the grid choice, a similar numerical analysis was carried out but using different grid spacings (2.5, 5, 7.5, 10, 25, 50, 75, and 100 ms). $t_{ce}$ and $t_{fe}$ values were varied between 2.5 and 505 ms, which led to 6 to 202 points for both $t_{ce}$ and $t_{fe}$ and grid sizes ranging from 36 to 40,804 total grid points. The boundary relationship between $t_{ce}$ and $t_{fe}$ was computed on each grid. RMSE on the testing set (15% points) as a function of the number of points along $t_{ce}$ is depicted in Figure A1. Noteworthy, as for grid spacings of 75 and 100 ms, using a 15% size for the testing set did not provide at least two points in such set. Therefore, two random points were forced to be attributed to the testing test (29 and 33% points in the testing set). As expected, RMSE decreased with decreasing grid spacing. Besides, it can be noticed that using a grid spacing of 10 ms did not seem to impact RMSE for the boundary relationship compared to the 7.5-ms grid spacing used before (RMSE ∼3.5 ms). However, the polynomial regression should also be performed on these different grids to observe any additional features.

For this reason, a multivariate polynomial regression (polynomial order from 1 to 15) was performed on 85% of the points composing these different grids, after having discarded the points which were not within the corresponding boundary relationship. RMSE on the testing set (15% points) as a function of grid size is depicted for each polynomial order in Figure A1. It can be noticed that the eighth-order polynomial is the lowest order polynomial, leading to an RMSE smaller than 0.5 ms on the testing set. In addition, the smallest grid to obtain such an RMSE threshold is given by a grid using a spacing of 7.5 ms, that is, 4,624 grid points. As for the grid spacing of 10 ms, it requires a polynomial of order 10 to achieve the requested RMSE threshold, which is less convenient as it requires 21 extra coefficients than the eighth-order polynomial. Therefore, these previous statements justify the grid choice used to construct the multivariate polynomial model (Eq. 3).

## References

[B1] Abendroth-Smith J. (1996). Stride Adjustments during a Running Approach toward a Force Plate. Res. Q. Exerc. Sport 67, 97–101. 10.1080/02701367.1996.10607930 8735999

[B2] Alcantara R. S. Day E. M. Hahn M. E. Grabowski A. M. (2021). Sacral Acceleration Can Predict Whole-Body Kinetics and Stride Kinematics across Running Speeds. PeerJ 9, e11199. 10.7717/peerj.11199 33954039 PMC8048400

[B3] Alexander R. M. (1989). On the Synchronization of Breathing with Running in Wallabies (Macropusspp.) and Horses (*Equus caballus*). J. Zoolog. 218, 69–85. 10.1111/j.1469-7998.1989.tb02526.x

[B4] Ammann R. Taube W. Wyss T. (2016). Accuracy of PARTwear Inertial Sensor and Optojump Optical Measurement System for Measuring Ground Contact Time during Running. J. Strength Conditioning Res. 30, 2057–2063. 10.1519/jsc.0000000000001299 26677827

[B5] Atkinson G. Nevill A. M. (1998). Statistical Methods for Assessing Measurement Error (Reliability) in Variables Relevant to Sports Medicine. Sports Med. 26, 217–238. 10.2165/00007256-199826040-00002 9820922

[B6] Backes A. Skejø S. D. Gette P. Nielsen R. Ø. Sørensen H. Morio C. (2020). Predicting Cumulative Load during Running Using Field‐based Measures. Scand. J. Med. Sci. Sports 30, 2399–2407. 10.1111/sms.13796 32767716

[B7] Bland J. M. Altman D. G. (1995). Comparing Methods of Measurement: Why Plotting Difference against Standard Method Is Misleading. The Lancet 346, 1085–1087. 10.1016/s0140-6736(95)91748-9 7564793

[B8] Brent R. P. (1973). Algorithms for Minimization without Derivatives. Englewood Cliffs, NJ: Prentice-Hall.

[B9] Broyden C. G. (1970). The Convergence of a Class of Double-Rank Minimization Algorithms 1. General Considerations. IMA J. Appl. Math. 6, 76–90. 10.1093/imamat/6.1.76

[B10] Camomilla V. Bergamini E. Fantozzi S. Vannozzi G. (2018). Trends Supporting the In-Field Use of Wearable Inertial Sensors for Sport Performance Evaluation: A Systematic Review. Sensors 18, 873. 10.3390/s18030873 PMC5877384 29543747

[B11] Cavagna G. A. Franzetti P. Heglund N. C. Willems P. (1988). The Determinants of the Step Frequency in Running, Trotting and Hopping in Man and Other Vertebrates. J. Physiol. 399, 81–92. 10.1113/jphysiol.1988.sp017069 3404473 PMC1191653

[B12] Cavagna G. A. Legramandi M. A. Peyré-Tartaruga L. A. (2008). Old Men Running: Mechanical Work and Elastic Bounce. Proc. R. Soc. B. 275, 411–418. 10.1098/rspb.2007.1288 PMC2596824 18077249

[B13] Cohen J. (1988). Statistical Power Analysis for the Behavioral Sciences. Oxfordshire, England, UK: Routledge.

[B14] Da Rosa R. G. Oliveira H. B. Gomeñuka N. A. Masiero M. P. B. Da Silva E. S. Zanardi A. P. J. (2019). Landing-takeoff Asymmetries Applied to Running Mechanics: A New Perspective for Performance. Front. Physiol. 10, 415. 10.3389/fphys.2019.00415 31040793 PMC6477028

[B15] Dalleau G. Belli A. Viale F. Lacour J. R. Bourdin M. (2004). A Simple Method for Field Measurements of Leg Stiffness in Hopping. Int. J. Sports Med. 25, 170–176. 10.1055/s-2003-45252 15088239

[B16] Debaere S. Jonkers I. Delecluse C. (2013). The Contribution of Step Characteristics to Sprint Running Performance in High-Level Male and Female Athletes. J. Strength Conditioning Res. 27, 116–124. 10.1519/jsc.0b013e31825183ef 22395270

[B17] Falbriard M. Meyer F. Mariani B. Millet G. P. Aminian K. (2018). Accurate Estimation of Running Temporal Parameters Using Foot-Worn Inertial Sensors. Front. Physiol. 9, 610. 10.3389/fphys.2018.00610 29946263 PMC6005819

[B18] Falbriard M. Meyer F. Mariani B. Millet G. P. Aminian K. (2020). Drift-Free Foot Orientation Estimation in Running Using Wearable IMU. Front. Bioeng. Biotechnol. 8, 65. 10.3389/fbioe.2020.00065 32117943 PMC7031162

[B19] Flaction P. Quievre J. Morin J. B. (2013). An Athletic Performance Monitoring Device. Washington, DC: U.S. Patent and Tradematk Office patent application.

[B20] Fletcher R. (1970). A New Approach to Variable Metric Algorithms. Comp. J. 13, 317–322. 10.1093/comjnl/13.3.317

[B21] Folland J. P. Allen S. J. Black M. I. Handsaker J. C. Forrester S. E. (2017). Running Technique Is an Important Component of Running Economy and Performance. Med. Sci. Sports Exerc. 49, 1412–1423. 10.1249/mss.0000000000001245 28263283 PMC5473370

[B22] Giandolini M. Horvais N. Rossi J. Millet G. Y. Samozino P. Morin J.-B. (2016). Foot Strike Pattern Differently Affects the Axial and Transverse Components of Shock Acceleration and Attenuation in Downhill Trail Running. J. Biomech. 49, 1765–1771. 10.1016/j.jbiomech.2016.04.001 27087676

[B23] Giandolini M. Poupard T. Gimenez P. Horvais N. Millet G. Y. Morin J.-B. (2014). A Simple Field Method to Identify Foot Strike Pattern during Running. J. Biomech. 47, 1588–1593. 10.1016/j.jbiomech.2014.03.002 24679708

[B24] Gindre C. Lussiana T. Hebert-Losier K. Morin J.-B. (2016). Reliability and Validity of the Myotest for Measuring Running Stride Kinematics. J. Sports Sci. 34, 664–670. 10.1080/02640414.2015.1068436 26177053

[B25] Goldfarb D. (1970). A Family of Variable-Metric Methods Derived by Variational Means. Math. Comp. 24, 23. 10.1090/s0025-5718-1970-0258249-6

[B26] Halilaj E. Rajagopal A. Fiterau M. Hicks J. L. Hastie T. J. Delp S. L. (2018). Machine Learning in Human Movement Biomechanics: Best Practices, Common Pitfalls, and New Opportunities. J. Biomech. 81, 1–11. 10.1016/j.jbiomech.2018.09.009 30279002 PMC6879187

[B27] Hébert-losier K. Mourot L. Holmberg H.-C. (2015). Elite and Amateur Orienteers' Running Biomechanics on Three Surfaces at Three Speeds. Med. Sci. Sports Exerc. 47, 381–389. 10.1249/mss.0000000000000413 24983340

[B28] Honert E. C. Mohr M. Lam W.-K. Nigg S. (2020). Shoe Feature Recommendations for Different Running Levels: A Delphi Study. PLOS ONE 15, e0236047. 10.1371/journal.pone.0236047 32673375 PMC7365446

[B29] Kram R. Dawson T. J. (1998). Energetics and Biomechanics of Locomotion by Red Kangaroos (*Macropus rufus*). Comp. Biochem. Physiol. B: Biochem. Mol. Biol. 120, 41–49. 10.1016/s0305-0491(98)00022-4 9787777

[B30] Lee J. B. Mellifont R. B. Burkett B. J. (2010). The Use of a Single Inertial Sensor to Identify Stride, Step, and Stance Durations of Running Gait. J. Sci. Med. Sport 13, 270–273. 10.1016/j.jsams.2009.01.005 19574098

[B31] Lussiana T. Patoz A. Gindre C. Mourot L. Hébert-Losier K. (2019). The Implications of Time on the Ground on Running Economy: Less Is Not Always Better. J. Exp. Biol. 222, jeb192047. 10.1242/jeb.192047 30787136

[B32] Lussiana T. Gindre C. (2016). Feel Your Stride and Find Your Preferred Running Speed. Biol. Open 5, 45–48. 10.1242/bio.014886 PMC4728304 26700723

[B33] Maiwald C. Sterzing T. Mayer T. A. Milani T. L. (2009). Detecting Foot-To-Ground Contact from Kinematic Data in Running. Footwear Sci. 1, 111–118. 10.1080/19424280903133938

[B34] Minetti A. E. (1998). A Model Equation for the Prediction of Mechanical Internal Work of Terrestrial Locomotion. J. Biomech. 31, 463–468. 10.1016/s0021-9290(98)00038-4 9727344

[B35] Moe-Nilssen R. (1998). A New Method for Evaluating Motor Control in Gait under Real-Life Environmental Conditions. Part 1: The Instrument. Clin. Biomech. 13, 320–327. 10.1016/s0268-0033(98)00089-8 11415803

[B36] Moore I. S. Ashford K. J. Cross C. Hope J. Jones H. S. R. Mccarthy-Ryan M. (2019). Humans Optimize Ground Contact Time and Leg Stiffness to Minimize the Metabolic Cost of Running. Front. Sports Act Living 1, 53. 10.3389/fspor.2019.00053 33344976 PMC7739683

[B37] Morin J.-B. Dalleau G. Kyröläinen H. Jeannin T. Belli A. (2005). A Simple Method for Measuring Stiffness during Running. J. Appl. Biomech. 21, 167–180. 10.1123/jab.21.2.167 16082017

[B38] Norris M. Anderson R. Kenny I. C. (2014). Method Analysis of Accelerometers and Gyroscopes in Running Gait: A Systematic Review. Proc. Inst. Mech. Eng. P: J. Sports Eng. Tech. 228, 3–15. 10.1177/1754337113502472

[B39] Novacheck T. F. (1998). The Biomechanics of Running. Gait & Posture 7, 77–95. 10.1016/s0966-6362(97)00038-6 10200378

[B40] Patoz A. Lussiana T. Thouvenot A. Mourot L. Gindre C. (2020). Duty Factor Reflects Lower Limb Kinematics of Running. Appl. Sci. 10, 8818. 10.3390/app10248818

[B41] Press W. H. Teukolsky S. A. Vetterling W. T. (1992). Numerical Recipes in FORTRAN: The Art of Scientific Computing. Cambridge, England: Cambridge University Press.

[B42] Purcell B. Channells J. James D. Barrett R. (2006). Use of Accelerometers for Detecting Foot-Ground Contact Time during Running. Proc. SPIE - Int. Soc. Opt. Eng. 6036, 292–299. 10.1117/12.638389

[B43] Shanno D. F. (1970). Conditioning of Quasi-Newton Methods for Function Minimization. Math. Comp. 24, 647. 10.1090/s0025-5718-1970-0274029-x

[B44] Smith L. Preece S. Mason D. Bramah C. (2015). A Comparison of Kinematic Algorithms to Estimate Gait Events during Overground Running. Gait & Posture 41, 39–43. 10.1016/j.gaitpost.2014.08.009 25212739

