# Supplementary material for: A Multivariate Polynomial Regression to Reconstruct Ground Contact and Flight Times Based on a Sine Wave Model for Vertical Ground Reaction Force and Measured Effective Timings
Source: Front Bioeng Biotechnol. 2021 Nov 4;9:687951. doi: 10.3389/fbioe.2021.687951 (PMC8599988; doi:10.3389/fbioe.2021.687951)
Supplement: Supplementary file 1 [file DataSheet1.PDF]

In[∘]:= Solve[Csc[(π * tg) / (tce + 2 * tg)] ==
π / 2 * ((tfe - 2 * tg) / (tce + 2 * tg) + 1), tg]

••• Solve: This system cannot be solved with the methods available to Solve.

Out[∘]= $\text{Solve}\left[\text{Csc}\left[\frac{\pi\,\text{tg}}{\text{tce} + 2\,\text{tg}}\right] == \frac{1}{2}\,\pi\left(1 + \frac{\text{tfe} - 2\,\text{tg}}{\text{tce} + 2\,\text{tg}}\right), \text{tg}\right]$
